# Parliament and the Professions

**Published:** 1919-03-29

**Authors:** 


### PARLIAMENT AND THE PROFESSIONS.

### Certified Assistants to Apothecaries.

Sir J. Agg-Gardner asked the Home Secretary whether he could state what action, if any, had been taken under the Poisons and Pharmacy Act, 1908, to enable certified assistants to apothecaries to be registered as pharmaceutical chemists or chemists and druggists. Mr. Shortt replied that the matter is now under the close consideration of the Pharmaceutical Society, and it is hoped that before long a by-law in the sense indicated will be submitted for the approval of the Privy Council.

### Demobilisation of Doctors and Nurses.

Mr. Churchill stated that he had given directions for a prompt and more general demobilisation of medical officers from the Royal Army Medical Corps, and that the rate of releases both of doctors and nurses had greatly increased. In the preceding week 700 doctors had been released.

### Cerebro-Spinal Fever.

Dr. Macnamara stated that there had been an outbreak of cerebro-spinal fever among young naval officers at Cambridge. Eight of fifty-four influenza cases in one ward of the No. 1 Eastern General Hospital developed cerebro-spinal fever. As the disease had not previously appeared in Cambridge and the district, it was apparently contracted from a "carrier." Two other cases were affected, the infection in these instances being traced. Of the ten cases five died, but every precaution has been taken, and the epidemic is considered to be well in hand.

### War Gratuities to Naval Medical Officers.

Dr. Macnamara informed Sir R. Woods that, following the practice of the Army, the war gratuities of temporary naval medical officers holding the rank of surgeon-lieutenant are at the rates of £60 and £50 for each year or part of a year's service for officers of the medical and dental branches respectively. The war gratuities of temporary medical officers above the rank of surgeon-lieutenant are based on their pay, and are on the same scale as those of other temporary officers, R.N.R. and R.N.V.R. The reason for the differentiation is that temporary surgeon-lieutenants are on special rates of pay, whilst those above that rank are on service rates.

### Influenza Hospitals Needed.

Major Astor, answering, on behalf of the President of the Local Government Board, questions as to the provision in isolation hospitals of accommodation for influenza patients landing from ships, stated that a conference has been arranged with representatives of the port sanitary authorities, at which this question, amongst others, will be discussed.

